# Retraction: Circular RNA hsa_circ_0072309 inhibits non-small cell lung cancer progression by sponging miR-580-3p

**DOI:** 10.1042/BSR-20194237_RET

**Published:** 2021-05-04

**Keywords:** hsa_circ_0072309, miR-580-3p, non-small cell lung cancer

This Retraction follows an Expression of Concern relating to this article previously published by Portland Press.

This article is being retracted from *Bioscience Reports* at the request of the Editor-in-Chief and the Editorial Board following receipt of a notification from a reader, alerting the Editorial Board to a duplicated image in Figure 4D.

The authors were contacted regarding the concerns raised and provided a replacement images for Figures 2D, 3F and 4D, however given that the replacement images for Figure 2D also contained a new duplicated image, the Editorial Board stands by the decision to retract. The authors were contacted in regards to the retraction but did not respond.

